# Subependymal Giant Cell Astrocytoma in an Adult without Tuberous Sclerosis: Systematic Review and Illustrative Case Example

**DOI:** 10.1055/a-2530-5965

**Published:** 2025-02-27

**Authors:** Brandon M. Holler, Alexander R. Evans, Abigail York, Christopher S. Graffeo

**Affiliations:** 1 Department of Neurosurgery, University of Oklahoma Health Sciences Center, Oklahoma City, Oklahoma, United States

**Keywords:** subependymal giant cell astrocytoma, tuberous sclerosis, systematic review

## Abstract

**Background** Subependymal giant cell astrocytoma (SEGA) is a rare neoplasm arising from subependymal tissue. Predominantly associated with the tuberous sclerosis complex (TSC), SEGA may present with a range of diverse symptoms, most commonly seizures or neurocutaneous features of TSC. We present a novel case of sporadic SEGA in a 59-year-old woman who presented with acute intraparenchymal hemorrhage (IPH).

**Methods** Systematic literature review and illustrative case example.

**Results** A 59-year-old woman presented with a headache decreased level of consciousness, and acute IPH involving the anterior septum pellucidum and right medial caudate head. MRI was concerning for an underlying neoplasm, which grew slowly on follow-up imaging, prompting microsurgical resection. A gross total resection was achieved, and postoperative pathology confirmed SEGA (WHO grade I) without *TSC1/2* mutation. She remained disease-free and neurologically intact at 1-year follow-up. A systematic review identified seven publications that revealed pathologically confirmed SEGA in nine adult patients without TSC. Headache, papilledema, and visual disturbances were the most common presenting symptoms. Treatment protocols included microsurgical resection versus biopsy followed by radiographic surveillance, and the overall rate of symptom-free survival was at least 80% as of the last follow-up.

**Conclusion** We report the tenth case of sporadic SEGA in an adult patient without TSC, as well as an associated systematic review of this rare neoplastic entity. Further study is required to identify risk factors for the development of sporadic SEGA, as well as potential avenues for the management of this disease that may depart from the standard protocol in pediatric TSC patients.

## Introduction

Subependymal giant cell astrocytoma (SEGA) is a rare neoplasm originating from the subependymal cells adjacent to the cerebral ventricles. Despite their benign nature (WHO grade I), these tumors have the capacity to precipitate a wide range of symptoms, ranging from hydrocephalus to cognitive impairment or seizures. SEGA is predominantly associated with the tuberous sclerosis complex (TSC), an autosomal dominant disease characterized by a characteristic series of neurocutaneous findings with variable penetrance such as seizures, hypomelanotic macules, and shagreen patches. 1 TSC almost universally presents in childhood or during prenatal screening, when more severe phenotypes may be diagnosed in the setting of cardiac rhabdomyomas or cortical tubers noted on gestational ultrasound. 2 Definitive diagnosis of TSC is defined by a mutation in either the *TSC1* or *TSC2* gene; however, the prognosis varies widely, ranging from mild symptoms to severe disability and a marked reduction in lifespan. 3 4 5 We report a unique case of a sporadic SEGA diagnosed in a 59-year-old woman who presented with acute intraparenchymal hemorrhage (IPH), and was subsequently confirmed to be negative for TSC.

## Methods

A literature search was conducted on October 2, 2023, across the PubMed, Google Scholar, and Ovid MEDLINE databases. Search terms included “SEGA” AND “without tuberous sclerosis” OR “in the absence of tuberous sclerosis.” Inclusion criteria included any original research article published in the English language detailing a case or case series of SEGA occurring without the presence of TSC in the adult population. Exclusion criteria included TSC-positive genetic results, patients under the age of 18, review papers, conference abstracts, letters to the editor, and studies published before the year 2000. Data extracted from each publication included sample size, age, biological sex, histopathological findings, presenting symptoms, clinical findings, and outcome.

## Results

### Illustrative Case Example

**Fig. 1:**
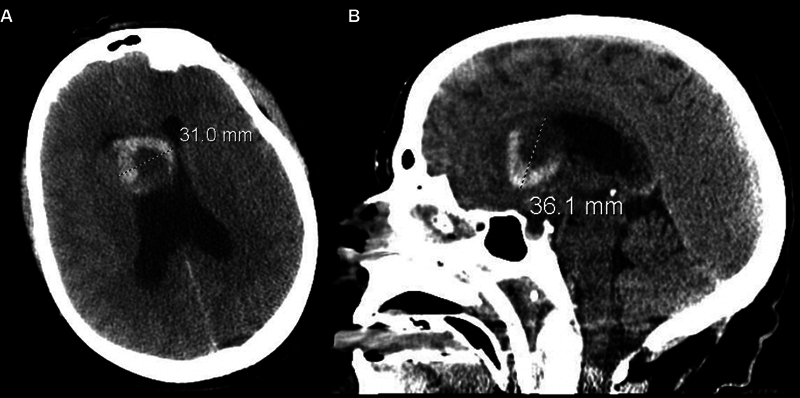
Initial axial (A) and sagittal (B) non-contrast CT head demonstrating acute intraparenchymal hemorrhage of the anterior septum pellucidum and right frontal lobe with intraventricular extension, measuring 31.0 mm by 36.1 mm in size.

**Fig. 2:**
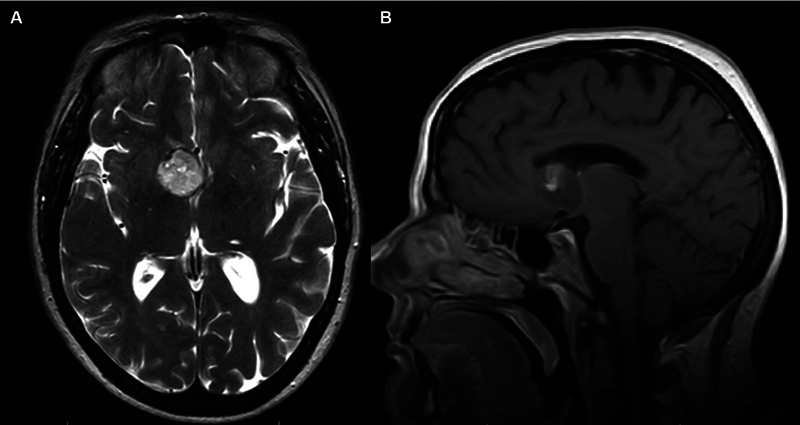
Contrast-enhanced T2-weighted axial (A) and T1-weighted sagittal (B) MRI brain suggestive of partially hemorrhagic neoplasm of the anterior septum pellucidum and right frontal lobe.

**Fig. 3:**
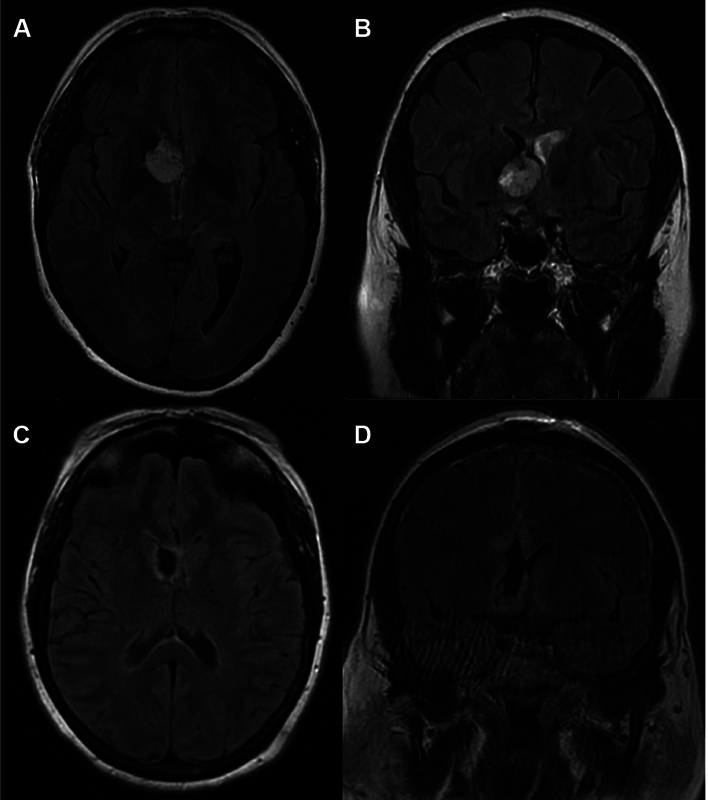
Preoperative axial (A) and coronal (B) contrast-enhanced T2-weighted FLAIR MRI demonstrating the 2.4 mm enhancing mass in the region of resolved right frontal periventricular hemorrhage. Also pictured: postoperative axial (C) and coronal (D) contrast-enhanced T2-weighted FLAIR MRI demonstrating the resection cavity following successful removal of the mass with residual T2/FLAIR hyperintensity most prominent along the posterior resection cavity margin.

A 59-year-old woman presented with headache and decreased level of consciousness, prompting head CT imaging and identification of an acute IPH with intraventricular hemorrhage (IVH) involving the anterior base of the septum pellucidum and the medial caudate head measuring 31.0 mm by 36.1 mm ( Fig. 1 ). CSF diversion was not required as the patient rapidly restored to her neurological baseline; however, diagnostic MRI showed a FLAIR-hyperintense mass without enhancement, concerning for an underlying neoplasm ( Fig. 2 ). This was initially followed, and when subtle interval growth was observed, microsurgical resection was recommended ( Fig. 3A, B ). The patient underwent left frontal craniotomy for anterior interhemispheric transcallosal approach and resection of the mass. Intraoperative frozen section was consistent with glioma, and so an aggressive resection was pursued. The patient recovered very well, neurologically intact at her preoperative baseline, and was discharged home on postoperative day 1, with a gross total resection confirmed on MRI ( Fig. 3C, D ). The final pathological diagnosis confirmed SEGA (WHO grade I), and the patient has continued to do well clinically and with no radiographic evidence of progression/recurrence as of her 1-year postoperative follow-up. Genetic testing indicated multiple tumor-specific mutations including whole-chromosome losses (3, 6, 10, 11, 13, 14, 17, 18, 21, and X) and point mutations in *TP53* , *NF1* , and *PTEN.* Germline mutation testing was within normal limits, including normal *TSC1/TSC2* .

### Literature Review

The systematic review identified 7 publications meeting the study criteria ( Table 1 ). The present case represents the 10th instance of a sporadic, pathological-confirmed SEGA diagnosed in an adult patient. Sex distribution was roughly even with a slight female predilection (56%); the mean age at presentation was 42 years (range: 20–75). Presenting symptoms were diverse and heterogeneous, with the most common being headache (40%), papilledema (20%), and visual disturbances (20%). Treatment protocols generally included elective resection or biopsy followed by radiographic surveillance clinical observation, and >80% of patients were alive and asymptomatic as of the last follow-up across all reported studies.

**Table 1:** Summary of adult cases of SEGA in the absence of tuberous sclerosis in the literature and the present case

Author	Sample size (sex)	Age a (y)	Genetic/histological findings	Presenting symptom(s)/clinical findings	Outcome
Stavrinou et al, 2008 6	1 (M)	33	SEGA identified histopathologically c	Headache, nuchal rigidity, bilateral papilledema	Surgical resection with postoperative external ventricular drain installation resulted in the resolution of symptoms
b Li et al, 2010 12	2 (M)	25	SEGA identified histopathologically c	Case 1: Bilateral mydriasis, papilledema, daytime sleepiness, fatigue, visual disturbance, vertigo, emesis Case 2: data not reported	Case 1: death secondary to massive L intraventricular hemorrhage Case 2: continued observation
Konakondla et al, 2016 7	1 (F)	25	Positive for GFAP and SYN; pathologically confirmed SEGA, WHO grade I, negative for *TSC1* and *TSC2*	Headache	Resection resulted in complete resolution of symptoms with no recurrence at 6 and 9 mo postoperatively
MacDonald et al, 2016 13	2 (M)	59.5 ± 9 (range: 53–66)	SEGA confirmed through surgical resection and stereotactic biopsy c	Case 1: headache, diplopia Case 2: generalized weakness secondary to diuretic-induced hypokalemia	Case 1: resection of mass with no recurrence at 13-mo postoperatively Case 2: continued observation
Takei et al, 2009 14	1 (F)	75	SEGA identified histopathologically c	Abdominal pain, dehydration, widespread metastasis of malignant melanoma d	Death d
Kashiwagi et al, 2000 15	1 (F)	20	Large gemistocytic cells with abundant cytoplasm and fibrillated spindle cells staining positive for GFAP and negative for S-100, NSE, NFL, and SYN	Headache	Resection resulted in complete resolution of symptoms with no recurrence at 28 mo postoperatively
Shelly et al, 2023 11	1 (F)	22	Negative for *TSC1* or *TSC2* ; SEGA identified histopathologically c	R ocular pressure, visual disturbance, pulsatile tinnitus	Neurologically intact following near-total resection; required ventriculoperitoneal shunt secondary to noncommunicating hydrocephalus postoperatively with no further complications
This study	1 (F)	59	Histologically confirmed WHO grade I SEGA; immunoreactive for GFAP and S-100; whole chromosome losses of 3, 6, 10, 11, 13, 14, 17, 18, 21, and X; variants in *TP53* , *NF1* , and *PTEN* genes; further genetic analysis for tuberous sclerosis pending	Headache and decreased level of consciousness secondary to acute intraparenchymal hemorrhage	Resection resulted in disease-free survival at 1 mo postoperatively

Abbreviations: F, female; GFAP, glial fibrillary acidic protein; L, left; M, male; NFL, neurofilament; NSE, neuron-specific enolase; R, right; SEGA, subependymal giant cell astrocytoma; SYN, synaptophysin.

a For studies with multiple patients, age is represented as mean ± standard deviation or median (range).

b Sex and age of the second case are not specified in the literature.

c Further genetic/histological findings were not reported.

d SEGA was identified incidentally upon autopsy as the patient presented with and succumbed to widespread metastatic melanoma.

## Discussion

We report a novel case of a sporadic SEGA in an adult without TSC, as well as a systematic literature review consolidating data together with the preceding nine cases. The present case is most unusual due to the presentation, which is relatively unique; two prior reports included IPH, but this is the first instance of IVH associated with sporadic SEGA in an adult patient. 6 7 Additionally, the molecular profile of the tumor is relatively atypical, including *TP53* and *PTEN* gene mutations that were previously unreported in SEGA diagnoses, 8 and only one prior case of NF1 mutation in a SEGA has been observed. 9 The unique features highlight an important area of insufficiency in our knowledge base regarding SEGA, and a potential avenue for future study exploring the relationships between SEGA, TSC, and other possible driver mutations.

Other key patterns from the small but focused literature on sporadic SEGA in adults include the relatively balanced gender distribution, and the prevalence of nonspecific symptoms generally referable to ICP elevation—headache, visual changes, and nausea or vomiting, in particular. 10 11 This emphasizes the difficulty of making rare diagnoses and the importance of a standardized protocol in assessing a tumor of unknown pathological origin. In this case, the indications for surgery included tumor growth on serial MRI and diagnostic uncertainty, with the possibility of a more aggressive entity such as low-grade glioma high on the differential. Needle biopsy was considered and was the initial treatment strategy of choice in several preceding cases; here, given the favorable location, the high probability prior to surgery that a definitive resection would be required, and the patient's expressed concern regarding additional hemorrhagic events and her stated preference for up-front resection rather than staged biopsy and possible resection prompted us to proceed directly to resection.

Although the knowledge base in this regard is clearly limited by the small available sample of extensively studied and reported cases, the current study does emphasize the possibility of a more complex constellation of genetic abnormalities underlying SEGA—typically considered a bland and benign diagnosis, especially beyond the considerations of TSC diagnoses. Indeed, the degree of chromosomal losses and genetic mutations noted in the current study may indicate a somewhat more aggressive phenotype, particularly given the subtle early expansion noted in tumor size. Further study will be required to better understand the implications of these findings, as well as the possible interplay between mutations in various tumor suppressor genes that are well-described outside the SEGA context— *PTEN* , *TP53* , and *NF1* . 9 In time, further case accumulation may reveal a stratified pathology, representing a range of possible mutations and associated prognostic or therapeutic implications.

### Limitations

The current study is subject to a range of limitations impacting essentially all observational studies, including those referable to small sample sizes and routine sources of bias. Additionally, the literature on sporadic SEGA in adults is widely distributed over time, with a high degree of variance in the extent of molecular results reported. Collectively, these cases provide a preliminary foundation, but amount to little more than anecdote and hypothesis-generating speculation regarding a rare and complicated diagnosis. Notwithstanding, the present case is novel and markedly enriches the limited available evidence regarding this rare neoplasm, highlighting new directions for possible future research.

## Conclusion

A novel case of sporadic SEGA is reported presenting with IPH/IVH in an adult patient without TSC, the tenth such tumor reported and the first to present in this fashion. Our study also identified a variety of novel genetic abnormalities of undetermined significance, but that may signal an increased risk of phenotypically aggressive tumor behavior that will require close follow-up in the future. Overall, SEGA remains an exceedingly rare diagnosis in the adult brain tumor population; however, the accumulation of additional cases involving this and other unusual pathologies is critical toward advancing future understanding and optimal treatment protocols.
